# Smart Thermometer–Based Participatory Surveillance to Discern the Role of Children in Household Viral Transmission During the COVID-19 Pandemic

**DOI:** 10.1001/jamanetworkopen.2023.16190

**Published:** 2023-06-01

**Authors:** Yi-Ju Tseng, Karen L. Olson, Danielle Bloch, Kenneth D. Mandl

**Affiliations:** 1 Computational Health Informatics Program, Boston Children’s Hospital, Boston, Massachusetts; 2 Department of Computer Science, National Yang Ming Chiao Tung University, Hsinchu, Taiwan; 3 Department of Pediatrics, Harvard Medical School, Boston, Massachusetts; 4 Kinsa Inc, San Francisco, California; 5 Department of Biomedical Informatics, Harvard Medical School, Boston, Massachusetts

## Abstract

**Importance:**

Children’s role in spreading virus during the COVID-19 pandemic is yet to be elucidated, and measuring household transmission traditionally requires contact tracing.

**Objective:**

To discern children’s role in household viral transmission during the pandemic when enveloped viruses were at historic lows and the predominance of viral illnesses were attributed to COVID-19.

**Design, Setting, and Participants:**

This cohort study of a voluntary US cohort tracked data from participatory surveillance using commercially available thermometers with a companion smartphone app from October 2019 to October 2022. Eligible participants were individuals with temperature measurements in households with multiple members between October 2019 and October 2022 who opted into data sharing.

**Main Outcomes and Measures:**

Proportion of household transmissions with a pediatric index case and changes in transmissions during school breaks were assessed using app and thermometer data.

**Results:**

A total of 862 577 individuals from 320 073 households with multiple participants (462 000 female [53.6%] and 463 368 adults [53.7%]) were included. The number of febrile episodes forecast new COVID-19 cases. Within-household transmission was inferred in 54 506 (15.4%) febrile episodes and increased from the fourth pandemic period, March to July 2021 (3263 of 32 294 [10.1%]) to the Omicron BA.1/BA.2 wave (16 516 of 94 316 [17.5%]; *P* < .001). Among 38 787 transmissions in 166 170 households with adults and children, a median (IQR) 70.4% (61.4%-77.6%) had a pediatric index case; proportions fluctuated weekly from 36.9% to 84.6%. A pediatric index case was 0.6 to 0.8 times less frequent during typical school breaks. The winter break decrease was from 68.4% (95% CI, 57.1%-77.8%) to 41.7% (95% CI, 34.3%-49.5%) at the end of 2020 (*P* < .001). At the beginning of 2022, it dropped from 80.3% (95% CI, 75.1%-84.6%) to 54.5% (95% CI, 51.3%-57.7%) (*P* < .001). During summer breaks, rates dropped from 81.4% (95% CI, 74.0%-87.1%) to 62.5% (95% CI, 56.3%-68.3%) by August 2021 (*P* = .02) and from 83.8% (95% CI, 79.2%-87.5) to 62.8% (95% CI, 57.1%-68.1%) by July 2022 (*P* < .001). These patterns persisted over 2 school years.

**Conclusions and Relevance:**

In this cohort study using participatory surveillance to measure within-household transmission at a national scale, we discerned an important role for children in the spread of viral infection within households during the COVID-19 pandemic, heightened when schools were in session, supporting a role for school attendance in COVID-19 spread.

## Key Points

**Question:**

What role did children play in household viral transmission during the COVID-19 pandemic, when enveloped virus rates were low and relative proportions of COVID-19 were at a high?

**Findings:**

In a cohort study of 166 170 households with adults and children using smart thermometers, among 38 787 inferred household transmissions over 3 years, 70.4% had a pediatric index case. Rates dropped during school breaks.

**Meaning:**

These results suggest that children were important viral vectors in households during the pandemic, particularly when school was in session.

## Teaser

This cohort study uses data from commercially available smart thermometers to estimate the role of children in household transmission of SARS-CoV-2 from the beginning of the pandemic until October 2022.

## Introduction

The role of children in viral spread during the SARS-CoV-2 pandemic has yet to be fully elucidated, but transmission within households plays an important role.^1,2,3,4,5,6,7^ Measurement traditionally requires labor-intensive and time-consuming contact tracing.^1,8,9^ We sought to discern within-household transmission using wide-area smart thermometer–based participatory surveillance. Participatory surveillance enables the public to report health-related information using the internet, smartphones, social media, and connected devices.^10,11^ It is a form of syndromic surveillance that relies on detection of clinical case features discernable before confirmed diagnoses are made.^12^ Participatory surveillance has been used to assess population health phenomena, providing a timely signal that complements traditional sources.^10,13^ We used a participatory surveillance cohort to study the role of children in within-household spread of virus during the COVID-19 pandemic, exploiting unique circumstances during a period when rates of enveloped viruses, such as influenza^14^ and respiratory syncytial virus (RSV),^15^ were at historical lows and the relative prevalence of viral illnesses attributed to COVID-19 was at a high.

## Methods

This is a retrospective cohort study of a nationwide cohort voluntarily using commercially available smartphone-connected thermometers (Kinsa Inc), who opted into data sharing using a companion app between October 1, 2019, and October 29, 2022. This time span was divided into pandemic periods^16,17,18^: before COVID-19 in the US (October 1, 2019, to February 29, 2020); first outbreak (March 1 to May 15, 2020); second period (May 16 to September 12, 2020); winter wave (September 13, 2020, to March 6, 2021); fourth period (March 7 to July 14, 2021); Delta wave (July 15 to December 18, 2021); Omicron BA.1/BA.2 wave (December 19, 2021, to June 19, 2022); and Omicron BA.4/BA.5 wave (June 20, 2022, to October 29, 2022). The Boston Children’s Hospital institutional review board found the study to be exempt, and informed consent requirements were waived because the study was secondary research using deidentified data. We followed the Strengthening the Reporting of Observational Studies in Epidemiology (STROBE) reporting guideline.

### Participants

Participants aged 18 years or older were considered adults. Children were grouped as younger (ages 0 to 8 years) or older (ages 9 to 17 years).^19^ The smartphone app let people share a thermometer but recorded data under unique user profiles. Thermometer data included temperature, body location where the temperature was taken, and a timestamp. Age and gender were self-reported. A household was defined as 1 or more individuals using the same thermometer or smartphone. Participants identified in more than 1 household were excluded. For analyses focused on household transmission, readings from a household with just 1 participant were excluded.

### Fever Definitions

Fever was defined as a temperature of at least 38.0 °C for rectal and aural readings, 37.8 °C for oral readings and readings from unknown body sites, and 37.2 °C for axillary readings.^20^ Temperature readings outside the range of 34 °C to 43 °C were excluded as outliers. Each participant’s readings were grouped into episodes that began after a period with no temperature measurements in the past 6 days. Fever onset in a febrile episode was defined as the first body temperature at or exceeding the limits described above.

### Transmission Inference

The index case was defined as the participant with the first fever onset in an inferred household transmission sequence. Secondary cases were defined as individuals with fever onset 1 to 7 days after the index case. Other fever transmissions were inferred community transmissions. If more than 1 participant fit the index case definition, the inferred transmission type was defined as unknown.

### Inferred Household Transmission Pattern Analysis

We analyzed inferred household transmission patterns in households with both adult and pediatric participants and compared patterns across pandemic periods, considering whether schools were in session or not. These patterns, starting with the index case, were classified as child to child, child to adult, adult to child, and adult to adult. We also analyzed differences when younger and older children were the index case.

### Statistical Analysis

Continuous variables were summarized as median values. Discrete variables were summarized as frequencies and percentages. All analyses were performed using R version 4.1.0 (R Foundation for Statistical Computing). All statistical tests were 2-sided, and statistical significance was defined as *P* < .05. The Granger causality test was used to measure the role of fever as a syndromic signature of viral infection (lmtest package). The Block bootstrap Mann–Kendall trend test was applied for trend detection (modifiedmk package).^21^ Significance for comparison of rates was tested with a Cochran-Armitage trend test (rstatix package). Spearman rank coefficient was calculated to assess the association between new COVID-19 cases and the proportion of inferred household transmissions with a pediatric index case.

## Results

### Population Characteristics

**Figure 1.**
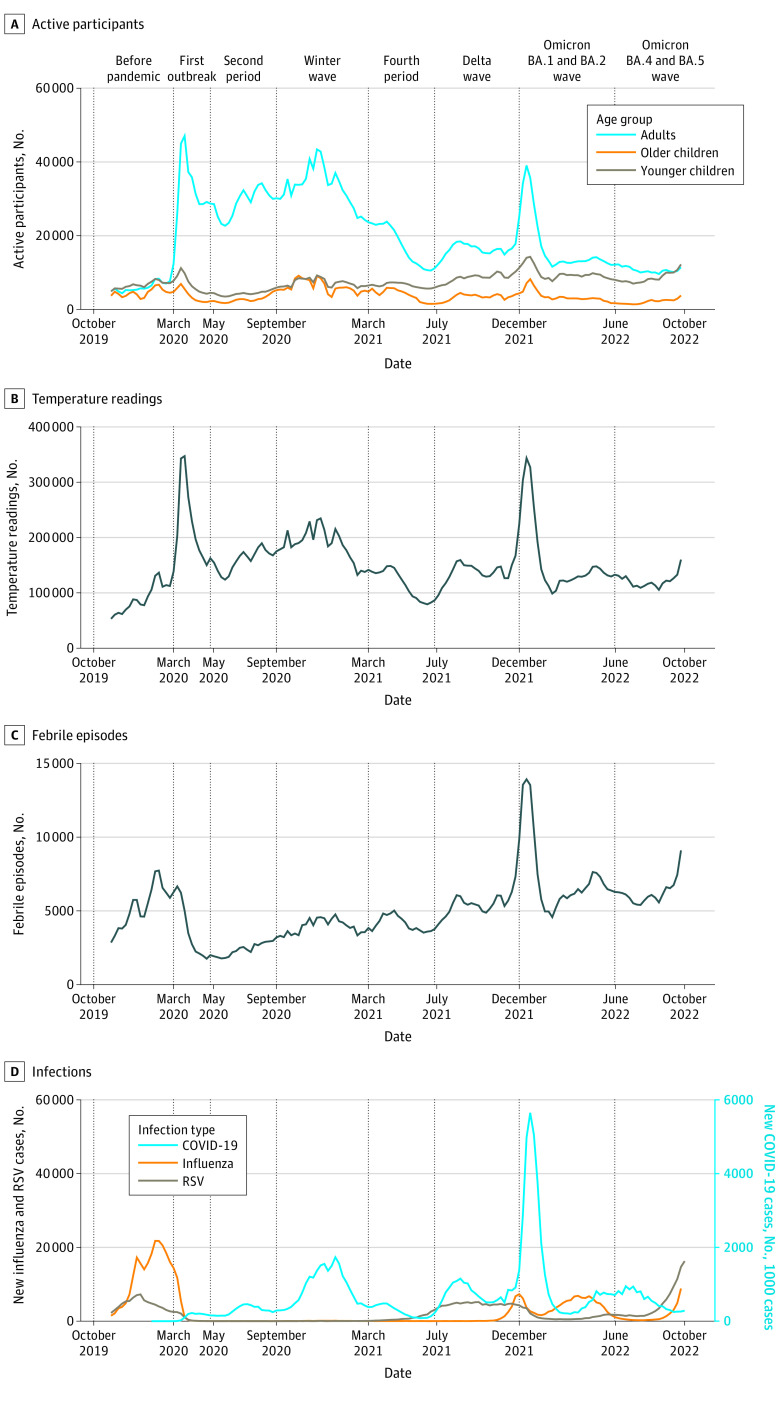
Time Series From Participatory Surveillance Data and Viral Case Rates From Publicly Available Data Sources Vertical lines define the beginning of each pandemic period. US COVID-19 case counts in panel D are sourced from the Johns Hopkins University Center for Systems Science and Engineering,^22^ the number of positive influenza cases from clinical laboratories using FluView Interactive,^14^ and the number of positive respiratory syncytial virus (RSV) cases from clinical laboratories in the National Respiratory and Enteric Virus Surveillance System (NREVSS).^15^

The 1 391 095 participants from 848 591 households took 23 153 925 temperature readings. More than half were from adults (803 116 [57.7%]), and more were from females than males for both adults (470 984 [58.6%] vs 291 871 [36.3%]) and children (284 582 [48.4%] vs 276 918 [47.1%]). There were 3 668 642 (15.8%) readings that met the criteria for fever, comprising 779 092 febrile episodes. The number of participants by age group, temperature readings, and febrile episodes per week are illustrated in Figure 1, along with new US COVID-19 case counts from the Johns Hopkins University Center for Systems Science and Engineering (JHU CSSE)^22^ and positive case counts for influenza^14^ and RSV.^15^ We found that number of febrile episodes forecast new COVID-19 cases (*F* = 20.0; *P* < .001), lending validity for using fever syndrome as a proxy for COVID-19 infection. Also, trends for temperature readings and active participants both peaked at the beginning of the pandemic and the Omicron wave.

In most households (528 518 [62.3%]), only 1 person shared temperature readings with the app. The remaining 320 073 households (37.7%) included multiple participants (862 577 individuals, 62.0% of all participants) taking 11 945 340 temperature readings (51.6% of all readings). The majority of children were younger (231 462 of 399 209 [58.0%]) and there were more female participants in each age group (Table 1).

**Table 1.** Characteristics of the Population in Households With 2 or More Participants

Characteristics	Children, No. (%)		Adults, No. (%)
	Age 0-8 y (younger)	Age 9-17 y (older)	
Participants	231 462 (26.8)	167 747 (19.4)	463 368 (53.7)
Age, median (IQR), y	4.0 (2.0-7.0)	12.0 (10.0-14.0)	38.00 (30.0-50.0)
Gender			
Female	111 124 (48.0)	83 341 (49.7)	267 535 (57.7)
Male	110 441 (47.7)	77 744 (46.3)	174 290 (37.6)
Other	906 (0.4)	672 (0.4)	2490 (0.5)
Unknown	8991 (3.9)	5990 (3.6)	19 053 (4.1)
No. of readings per participant, median (IQR)	5.0 (2.0-15.0)	3.0 (1.0-10.0)	3.0 (1.0-11.0)
No. of episodes per participant, median (IQR)	2.0 (1.0-3.0)	1.0 (1.0-2.0)	1.0 (1.0-3.0)
Household composition			
Adults and children	147 305 (63.6)	117 888 (70.3)	250 891 (54.1)
Adults only	NA	NA	212 477 (45.9)
Children only^a^	84 157 (36.4)	49 859 (29.7)	NA

^a^ Households with children only may have had adults in the household who were not using the thermometer.

### Household Transmission

**Figure 2.**
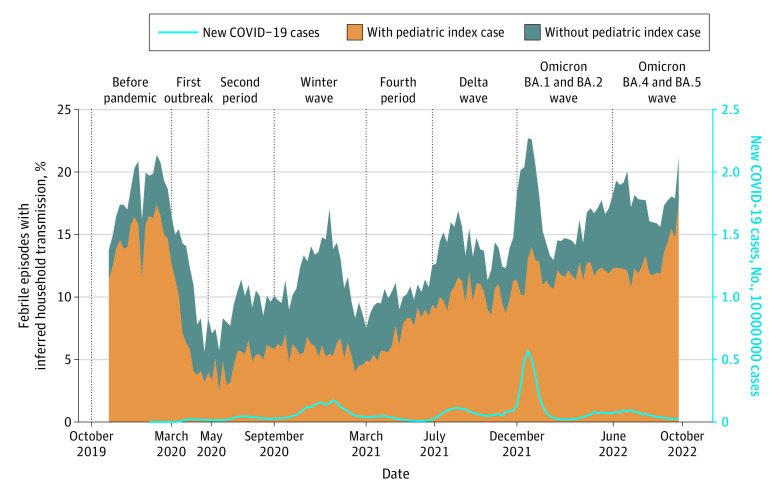
Inferred Household Transmissions Among All Febrile Episodes in Households With Multiple Participants With or Without a Pediatric Index Case Vertical lines define the beginning of each pandemic period.

There were 354 602 febrile episodes in households with multiple participants. Of these, 54 506 (15.4%) were considered inferred household transmissions. The percentage of inferred household transmissions increased from the fourth period (3263 of 32 294 [10.1%]) to the Omicron BA.1/BA.2 wave (16 516 of 94 316 [17.5%]) (*P* < .001) (Figure 2).

### Inferred Household Transmission Patterns

There were 166 170 households with both adult and child participants (51.9% of households with multiple participants). These households included 516 159 participants, 265 268 (51.4%) children under 18 years old, who took 6 227 726 temperature readings. The 38 787 inferred household transmissions occurred with the following patterns: 15 819 (40.8%) were child to child, 11 481 (29.6%) child to adult, 7865 (20.3%) adult to child, and 3622 (9.3%) adult to adult. The median (IQR) serial interval between the index and secondary cases was 2 days (IQRs of 1-3 for adult to adult and child to child in the second period, child to child in the winter wave, and adult to adult and adult to child in the Omicron wave; other intervals were 1-4) across all the pandemic periods and transmission patterns, except for the first outbreak where child to child and child to adult was 3 days (1-4 days).

**Figure 3.**
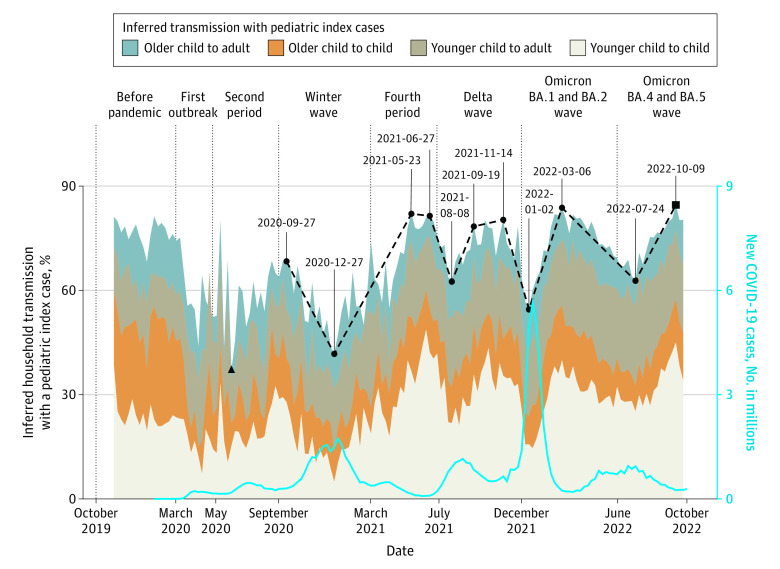
Patterns of Inferred Household Transmissions With a Pediatric Index Case Lowest and highest percentages during the pandemic are highlighted with black triangle and square points, respectively. Vertical lines define the beginning of each pandemic period. The blue line is the number of new US COVID-19 cases. The points with dates are relatively high and low levels of pediatric transmissions and the dashed line is the trend with these points.

Among all inferred household transmissions, 27 300 (70.4%) started with a pediatric index case but this proportion fluctuated weekly between a low of 36.9% and a high of 87.5% (median [IQR], 70.4% [61.4%-77.6%]) (Figure 3). During all but the second pandemic period, as well as across the whole study period, the percentage of pediatric transmissions was negatively correlated with new COVID-19 cases (Table 2).

**Table 2.** Correlation Between Number of New COVID-19 Cases per Week and the Proportion of Inferred Household Transmissions With a Pediatric Index Case

Period	No. of wks	Spearman ρ	*P* value
First outbreak	11	−0.627	.04
Second period	17	−0.145	.58
Winter wave	25	−0.667	<.001
Fourth period	19	−0.877	<.001
Delta wave	22	−0.474	.03
Omicron BA.1/BA.2 wave	27	−0.875	<.001
Omicron BA.3/BA.4 wave	18	−0.944	<.001
Whole study period	145	−0.340	<.001

We tracked changes in transmission rates by dates corresponding to the beginning and end of the school years and timing of the winter breaks (Figure 3). Pediatric transmissions started at a high of 68.4% (95% CI, 57.1%-77.8%) during the week of September 27, 2020, and declined to a low of 41.7% (95% CI, 34.3%-49.5%) during the week of December 27, 2020 (0.61 times less frequent; *P* < .001). The next high point was 82.0% (95% CI, 74.3%-87.8%) during the week of May 23, 2021, which remained stable until June 27, 2021 (81.4%; 95% CI, 74.0%-87.1%). A decrease to 62.5% (95% CI, 56.3%-68.3%) followed by August 8, 2021 (0.77 times less frequent; *P* = .007). By September 19, 2021, the percentage had increased again to 78.4% (95% CI, 73.4%-82.6%; *P* = .02) and remained stable until November 14, 2021 (80.3%; 95% CI, 75.1%-84.6%). The next low was 54.5% (95% CI, 51.3%-57.7%) the week of January 2, 2022 (0.68 times less frequent; *P* = .009). The next increase was to 83.8% (95% CI, 79.2%-87.5%) by March 6, 2022, followed by a decrease to 62.8% (95% CI, 57.1%-68.1%) in the week ending July 24, 2022 (0.75 times less frequent; both *P* < .001). The final high point of 84.6% (95% CI, 80.6%-87.8%) was during the week of October 9, 2022 (*P* < .001).

### Pediatric Index Cases

Pediatric index cases were examined in 2 age groups, younger (age 0 to 8 years) and older (age 9 to 17 years) (Figure 3). Younger children (17 572, 7.6% of all younger children) were more likely to be the index cases for an inferred household transmission than older children (9,728, 5.8% of all older children) (*P* < .001).

## Discussion

At a time when the relative rate of viral illnesses attributable to COVID-19 was at a high, we were able to take advantage of participatory surveillance methods to gain insight into transmission dynamics among 1.4 million individuals in over 800 000 households using commercially available shared thermometers and a smartphone app. Our findings suggest that children play an important role in within-household viral transmissions. Consistent with demonstrated patterns among other viral illnesses, pediatric-driven transmission was higher when school was in session. During the COVID-19 pandemic, inferred household transmissions increased from the fourth pandemic period (March 7 to July 14, 2021) to the Omicron BA.1/BA.2 wave. More than 70% of household transmissions in households with adults and children were from a pediatric index case, but this percentage fluctuated weekly. Once US schools reopened in fall 2020,^23,24,25^ children contributed more to inferred within-household transmission when they were in school, and less during summer and winter breaks, a pattern consistent for 2 consecutive school years.

In over 166 000 households with both adults and children, where over 6 million temperature readings were recorded, we found that children represented the majority of index cases after schools reopened in both the 2020-2021 and 2021-2022 school years. However, these transmissions decreased during summer and winter school breaks, which is consistent with prior studies showing school attendance associated with increased respiratory viral spread, and school holidays with decreased spread.^26,27,28^ While it is known that children play an important role in the spread of respiratory viruses,^5,29,30,31^ their contribution to the transmission of SARS-CoV-2 has been unclear.^1,2,3,4,5^ The heterogeneity of prior findings on this topic could be due to several factors including relatively small numbers of households studied, limitations of traditional observational studies,^32^ and features of the different pandemic periods such as variant type, school policy, and availability of vaccines. In the early pandemic months, school closure was common across the globe,^33,34^ limiting school transmissions and making children far less important as drivers of SARS-CoV-2 transmission than adults.^2,35^ However, once schools reopened in fall 2020,^23,24,25^ children could interact more with others in the community. The number of pediatric COVID-19 cases increased, with evidence that this increase affected overall spread.^2,5,6,9,36,37^ During the winter wave, children in England were more likely to introduce the virus to households than adults.^32^ Transmissions from pediatric index cases to household contacts were frequent in California^38,39,40^ and Colorado.^40^ During the Delta wave, children were more likely to transmit infection in households in Singapore.^41^ Consistent with our findings, these studies suggest that the role of children in household transmission became important after school reopened, and changed over time. Our finding that there was more within-household transmission during the Omicron wave is consistent with previous studies.^37,42,43^

After the winter wave, the percentage of inferred household transmissions with a pediatric index case was negatively correlated with the number of new COVID-19 cases. This is consistent with a previous study showing that during a period of low community transmission, children were the predominant index cases, while during a high community transmission period, adults were.^39^ Other investigators have shown that the risk of SARS-CoV-2 infection in educational settings correlates with community infection rates,^44,45^ and that spread among children in school was lower than among adults in the community.^46^ When the incidence of COVID-19 increases, adults in the community are at higher risk of infection; this may increase the likelihood that adults become the index case in a household transmission and explain the negative correlation we observed. Also, when the COVID-19 incidence is low, overall use of nonpharmaceutical interventions might decrease, leading to increased incidence of non–SARS-CoV-2 pathogens which may be more common in children.

The measurement technique used in this investigation is a form of syndromic surveillance, an approach developed over 2 decades ago.^12^ The frequency of fevers reported via smartphone-connected thermometers was predictive of population level COVID-19 case counts. This relationship serves to further validate fever measured at home as a syndromic surveillance data source for infectious disease monitoring. In previous studies, actual temperature readings from smart thermometers have been used to detect COVID-19 epicenters,^47^ and forecast influenza^48^ and influenza-like illnesses.^20^ During the COVID-19 pandemic, various citizen-facing participatory surveillance systems were rapidly deployed and used by millions of people.^49,50,51,52,53,54^ Online surveys underpinned a large remote global health monitoring system and were used for tracing COVID-19 impact trajectories^52^ and deriving household COVID-19 risk.^55^ Participatory surveillance systems provide complementary information to the traditional surveillance system and can be an important source of real-time data.^13,49,56,57,58^ Furthermore, they can capture information about conditions that do not result in a health care visit and its subsequent medical record or insurance claim.

### Limitations

This study had several limitations. The study design did not permit laboratory or home testing to confirm viral etiologies. Fever as a syndrome has many etiologies beyond COVID-19. Although confirmatory tests are needed to definitively identify the origin of fever, our study exploited a unique period when the incidence of generally prevalent, non–COVID-19 respiratory viruses plunged, including influenza^14^ and RSV.^15^ Although non–SARS-CoV-2 viruses were circulating, we assumed their prevalence was comparatively limited during the study period, with the number of COVID-19 cases^22^ during the study period being 10 to over 100 times higher than that of influenza^14^ and RSV^15^ until August 2022. Parainfluenza^59^ and human metapneumovirus^59^ were also rare. Among patients with symptoms hospitalized or presenting to the emergency department (ED), the incidence of rhinoviruses and enteroviruses dropped at the beginning of the pandemic until October 2020, but rose between then and February 2021.^59^ However, in a community-based study perhaps more reflective of the epidemiology of our home-based cohort, SARS-CoV-2 was more common than rhinovirus from December 2021 to July 2022.^60^ COVID-19 has been reported to be of very high incidence,^22^ although generally mild in children,^61,62,63^ who may be asymptomatic and not develop a fever.^64^ Given the wide availability of rapid testing at home by December 2020,^65^ many children with COVID-19 may not have had medical system encounters. We suspect that using rates of children presenting to the ED or hospital with a positive polymerase chain reaction test may underestimate the incidence of COVID-19. Nonetheless, COVID-19 was one of the highest reasons for pediatric ED visits until January 2022, and compared with 2019, visits for non–COVID-19 respiratory illness declined in 2020 and 2021.^66^ Reassuringly, the number of fevers measured in our cohort was a useful indicator of population-level COVID-19 case counts, supporting our contention that the measured household transmission patterns were reflective of COVID-19 dynamics. Notably, fever patterns also accurately forecasted influenza,^20,48^ even when other viruses were circulating. A fever-based monitoring approach may, in fact, underestimate COVID-19 rates. Prior work has shown that symptoms varied depending on vaccine status and SARS-CoV-2 variant, with fever less common during the Omicron wave.^67,68,69^ Changes in age-related presentations varied across studies. One US study^69^ concluded that percentages of fever among children under age 12 years, adolescents (ages 12 to 18 years), and adults (older than 18 years) were not different among symptomatic individuals testing positive during the Omicron BA.1 period. However, a United Kingdom study^68^ found that fever was more common in children experiencing symptoms and with positive polymerase chain reaction tests who were younger than 5 years during the Omicron BA.1 and BA.2 periods. During the Omicron waves, children and adolescents had a higher proportion of asymptomatic infection.^70^

Recruitment and retention of participants that reflect the population is a common challenge for participatory surveillance.^71^ Structural bias related to access to digital technologies can skew results by socioeconomic and racial factors. Note that an alternative method, contact tracing, may also produce a selection bias, and it cannot achieve the scale of this study. In addition, some groups could have more temperature readings than others. Perhaps parents are more likely to take their children's temperatures than their own, thus overestimating proportions with a pediatric index case. However, in this study, there were more temperature readings per person for adults than children. Antipyretic use could also have affected results, although the thermometers used should have been able to capture the initial fever signal before antipyretic use began. Furthermore, we defined a household as people using the same thermometer or smartphone to report temperature readings. However, there could be other people in the same household who used a different thermometer, did not measure their body temperature, or did not opt into data sharing.

Identifying the transmission chain has challenges, even with detailed conventional epidemiological investigations. Secondary cases could be from outside exposure rather than an infected household member. However, by restricting occurrence to within 1 week after the index case, we sought to mitigate this issue. The median serial interval between the index and secondary case was 2 days.

The quality of self-measured data, even with an FDA-cleared device, may be lower than that collected by a health professional. However, the real-time availability of these data has proven useful for identifying areas that need COVID-19 tests,^57^ documenting symptoms,^13,49,56^ forecasting epidemics,^72^ tracking the spread of infections,^56^ and identifying disease activity change weeks ahead of traditional disease surveillance.^73^

Vaccinated individuals may experience mild symptoms,^69^ so it is possible that the contribution of adults was underestimated due to higher vaccination coverage. Yet, a decreasing trend in the proportion of household transmissions with a pediatric index case was observed during the winter break at the end of 2020, before the initial vaccination protocol was widely completed.^74^ The companion app used in our study allowed participants to report other symptoms, which could have served as additional indicators if fever became less common and other symptoms became more common. An additional survey could be distributed through the app to collect vaccine status and other information.

A potential confounder is that nonpharmaceutical interventions changed over time and varied across states. Not all schools were open for in-person instruction in fall 2020. About 19% of K-12 schools remained fully online and 50% were using a hybrid model.^23,24^ In a manual review of 250 school districts, 29% never opened in-person during fall 2020.^25^ Therefore, the degree to which school breaks in 2020 factored into our results may be underestimated. However, as time went on, more schools chose to reopen for in-person classes.^23,24^ In the spring and fall 2021, very few school districts, only 2% and 0.5%, respectively, remained fully online.^24^ The percentage of schools requiring masks decreased from 68% to 7% between fall 2020 and spring 2022.^24^ In fall 2020, when schools had just reopened, 68.4% of inferred household transmissions started with a pediatric index case. In fall 2021 and 2022, 78.4% and 84.6% did. These latter 2 school years coincided with more in-person classes and fewer requirements for masks and other nonpharmaceutical interventions.

## Conclusion

In this cohort study using participatory surveillance to measure within-household viral transmission, children played an important role in viral spread during the SARS-CoV-2 pandemic, at a time when the relative incidence and prevalence of COVID-19 was maximal compared with other circulating viruses. Pediatric transmission was heightened when school was in session and when community levels of COVID-19 were lower, suggesting a substantial role of school attendance in COVID-19 spread. Surveillance with smartphone connected devices enabled infectious disease surveillance and measurement of infection dynamics at a scale unobtainable with traditional methods. The approach brings research into the household without needing study personnel or contact tracers. Future work could validate the inferred transmissions from a participatory network with onsite visits or other contract-tracing outreach for additional data collection and laboratory confirmation. Any system that leverages digital technologies must make every effort to ensure equitable access.

## Notes

Supplement.
Data Sharing Statement
