# Error in Results

**DOI:** 10.1001/jamanetworkopen.2023.30424

**Published:** 2023-08-11

In the Original Investigation titled “Smart Thermometer–Based Participatory Surveillance to Discern the Role of Children in Household Viral Transmission During the COVID-19 Pandemic,”^1^ published June 1, 2023, total individuals in the surveillance network were used when the households with adults and children were more relevant to study results. In the Results, the total number of participants has been changed from 516 159 to 516 084, and total children from 265 268 to 265 193; percentages of younger and older children that were index cases of households have been updated with 147 305 younger and 117 888 older children as the denominators, respectively. Additionally, language in the Key Points, Discussion, and Conclusion has been updated to clarify the study population. This article has been corrected.^1^
