# Supplementary material for: Smart Thermometer–Based Participatory Surveillance to Discern the Role of Children in Household Viral Transmission During the COVID-19 Pandemic
Source: JAMA Netw Open. 2023 Jun 1;6(6):e2316190. doi: 10.1001/jamanetworkopen.2023.16190 (PMC10236238; doi:10.1001/jamanetworkopen.2023.16190)
Supplement: Supplement. — Data Sharing Statement [file jamanetwopen-e2316190-s001.pdf]

Data Sharing Statement

Tseng. Smart Thermometer-Based Participatory Surveillance to Discern the Role of Children in Household Viral Transmission During the COVID-19 Pandemic. *JAMA Netw Open*. Published June 01, 2023. doi:10.1001/jamanetworkopen.2023.16190

Data
Data available: Yes
Data types: Deidentified participant data
How to access data: Kinsa Inc will make data available to others upon request and upon completion of a data use agreement, only for research, and non-commercial purposes to individuals affiliated with academic or public health institutions.
When available: With publication

Supporting Documents
Document types: None

Additional Information
Who can access the data: Kinsa Inc will make data available to others upon request and upon completion of a data use agreement, only for research, and non-commercial purposes to individuals affiliated with academic or public health institutions.
Types of analyses: Only for research, and non-commercial purposes to individuals affiliated with academic or public health institutions
Mechanisms of data availability: After completion of a data use agreement
